# Resistivity dependence of magnetoresistance in Co/ZnO films

**DOI:** 10.1186/1556-276X-9-6

**Published:** 2014-01-06

**Authors:** Zhi-Yong Quan, Li Zhang, Wei Liu, Hao Zeng, Xiao-Hong Xu

**Affiliations:** 1 Key Laboratory of Magnetic Molecules and Magnetic Information Materials of Ministry of Education and School of Chemistry and Materials Science, Shanxi Normal University, Linfen 041004, China; 2 Department of Physics, University at Buffalo, the State University of New York, Buffalo, NY 14260, USA

**Keywords:** ZnO, Magnetoresistance, Resistivity, Tunneling, Higher-order hopping, 77.55.hf, 75.47.De, 75.45. + j

## Abstract

We report the dependence of magnetoresistance effect on resistivity (*ρ*) in Co/ZnO films deposited by magnetron sputtering at different sputtering pressures with different ZnO contents. The magnitude of the resistivity reflects different carrier transport regimes ranging from metallic to hopping behaviors. Large room-temperature magnetoresistance greater than 8% is obtained in the resistivity range from 0.08 to 0.5 Ω · cm. The magnetoresistance value decreases markedly when the resistivity of the films is less than 0.08 Ω · cm or greater than 0.5 Ω · cm. When 0.08 Ω · cm < *ρ* < 0.5 Ω · cm, the conduction contains two channels: the spin-dependent tunneling channel and the spin-independent second-order hopping (*N* = 2). The former gives rise to a high room-temperature magnetoresistance effect. When *ρ* > 0.5 Ω · cm, the spin-independent higher-order hopping (*N* > 2) comes into play and decreases the tunneling magnetoresistance value. For the samples with *ρ* < 0.08 Ω · cm, reduced magnetoresistance is mainly ascribed to the formation of percolation paths through interconnected elongated metallic Co particles. This observation is significant for the improvement of room-temperature magnetoresistance value for future spintronic devices.

## Background

The investigation of electron spin transport from metallic ferromagnets to semiconductors has been an active research field in spintronics in the past two decades [1-3]. The manipulation of carrier spins between magnetic metals and semiconductors provides improved functionality of spintronic devices such as magnetic sensors, spin transistors, and magnetic memory cells [4,5]. Spin injection into a semiconductor reveals low efficiency in ferromagnetic metal/semiconductor films at room temperature (RT) because of a significant mismatch in conductivities [6-8].

Recently, magnetic metal/semiconductor films have been considered for their large magnetoresistance (MR) at RT, which is responsible for effective spin injection into semiconductors [9-14]. However, the origin of MR and the different influential factors for the MR effect are controversial. Yan et al. reported a large negative MR of 11% at RT in Co/ZnO films, which was ascribed to spin-dependent variable range hopping [9]. Hsu et al. observed transverse magnetotransport transition from a negative MR of 4.6% to the anomalous Hall effect at RT and found a variation with different annealing temperatures in a Co/ZnO film [10]. In our previous publications, we obtained a larger RT MR ratio of approximately 12.3% in a Co/ZnAlO granular film that resulted from spin-dependent tunneling through semiconductor barriers and observed that the values of MR changed with the film thickness in Co/ZnO granular films [12,13]. By contrast, Varalda et al. investigated Fe/ZnSe films consisting of Fe-clustered particles embedded in a ZnSe matrix and observed significant negative MR only at low temperature [15]. These inconsistent results may likely be attributed to the fact that the MR effect of magnetic metal/semiconductor films is extremely sensitive to fabrication conditions resulting in varied microstructures and defects in semiconductors. However, up to now, few experiments have been performed for the systematic study to correlate these structural properties with magnetotransport. Besides, the mechanism of MR remains unclear. Thus, investigating this issue may help us better understand the physics involved and achieve a higher MR ratio at higher temperature for practical applications.

In this work, we studied a large number of Co/ZnO films deposited at different sputtering pressures with different ZnO thicknesses and found that the MR effect is strongly dependent on the resistivity of films. We further investigated the charge transport in these films and found that conduction can be separated into three regimes, namely metallic, tunneling, and hopping regimes, with different temperature dependence. We found that among the three regimes, only the tunneling part is strongly spin dependent. This leads to a broad maximum of MR in the tunneling regime. This finding is useful in the tuning of MR values and in understanding its mechanism.

## Methods

Co/ZnO films were deposited by sequentially sputtering ultrathin Co layers and ZnO layers on glass substrates at RT. Direct-current and radio-frequency powers were applied to Co and ZnO targets, respectively. The sputtering chamber pressure was reduced to 8 × 10^−5^ Pa before deposition. The sputtering gas was an Ar atmosphere with a range of 0.4 to 0.8 Pa. The film nominal structure is [Co (0.6)/ZnO (*x*)]_60_ (denoted as Co/ZnO; thicknesses in nanometers), where *x* = 0.3 to 2.5 nm is the thickness of the ZnO layer. The details of the growth have been described in a previous publication [11].

The thickness of the films was measured by a surface profiler. The structures of the films were analyzed using X-ray diffraction (XRD). The magnetic properties of the films were measured using a superconducting quantum interference device magnetometer with a magnetic field applied parallel to the film plane. The magnetic field dependence of MR was measured using a conventional four-probe method in the maximum applied magnetic field of 20 kOe with current in the plane at RT. The temperature dependence of resistance was measured by four-point geometry from 5 to 300 K.

## Results and discussion

**Figure 1:**
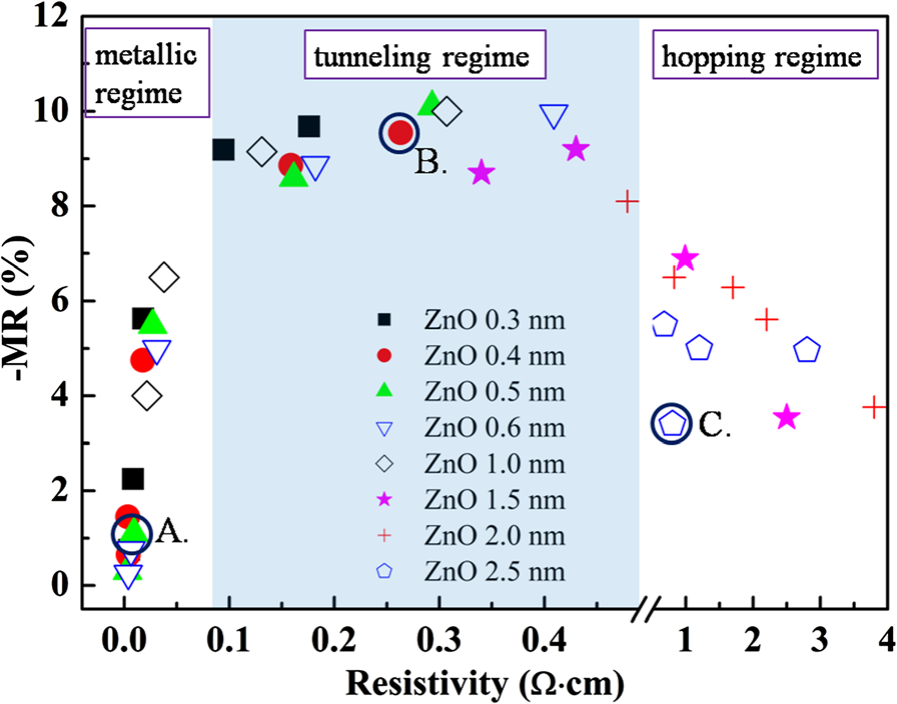
**MR value of Co/ZnO films as a function of resistivity.** We fixed the composite of Co/ZnO films and varied sputtering pressures from 0.4 to 0.8 Pa; we also fixed the sputtering pressure and changed the film thickness of the ZnO layer from 0.3 to 2.5 nm. Samples A, B, and C, labeled as solid circles, are situated in the metallic, tunneling, and hopping regimes, respectively.

The key result of our work is presented in Figure 1, which clearly shows that the RT MR is strongly correlated with resistivity and therefore the transport behavior of Co/ZnO films. We found that the reproducibility of the films was very good and that there is no clear correlation between the ZnO thickness, the chamber sputtering pressure, and the values of MR. However, a clear pattern emerges when MR is plotted against the resistivity of the films. From Figure 1, the MR values are evidently larger than 8.1% in the intermediate regime (tunneling regime) with 0.08 Ω · cm < *ρ* < 0.5 Ω · cm, but they decrease markedly in the left and right regimes (metallic and hopping regimes). In the metallic regime, the MR effect becomes weaker with decreasing resistivity and finally trends toward zero as the resistivity decreases to approximately 0.004 Ω · cm. The MR also decreases with increasing resistivity in the hopping regime and retains at 3.7% when the resistivity reaches approximately 3.8 Ω · cm in the hopping regime, as shown in Figure 1.

**Figure 2:**
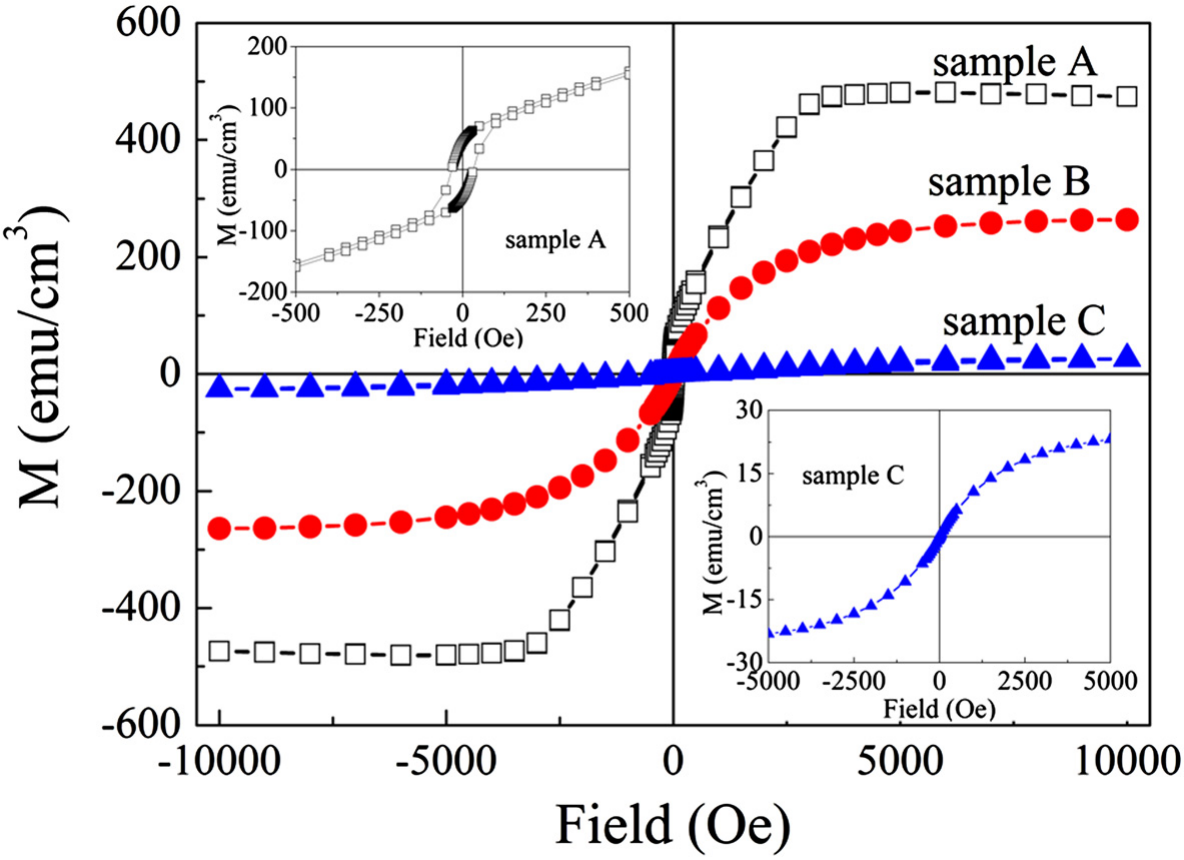
**Hysteresis loops of three Co/ZnO films: samples A, B, and C at RT.** The two insets show the enlarged loops of samples A and C.

To investigate the mechanisms behind the dependence of MR on resistivity, we selected three typical samples: Co/ZnO films with *x* = 0.5 sputtered at 0.4 Pa (marked as sample A), *x* = 0.4 sputtered at 0.8 Pa (marked as sample B), and *x* = 2.5 sputtered at 0.8 Pa (marked as sample C) (shown in Figure 1). Figure 2 shows the hysteresis loops of the three films measured with a magnetic field applied to the film plane at RT after subtracting the diamagnetic background. The magnetization curves of samples B and C exhibit a superparamagnetic-like nature, with negligible remanence and coercivity. This indicates that Co nanoparticles may exist in the films. Whereas, as shown in the inset of Figure 2, a coercivity value of 34 Oe is observed in sample A, which may be attributed to the formation of interconnected large Co particles in the films. The saturation magnetization decreases from 476 to 264 and 25 emu/cm^3^ for samples A, B, and C, respectively. This decrease may be attributed to the decreasing size of Co particles and the increasing ZnO content.

**Figure 3:**
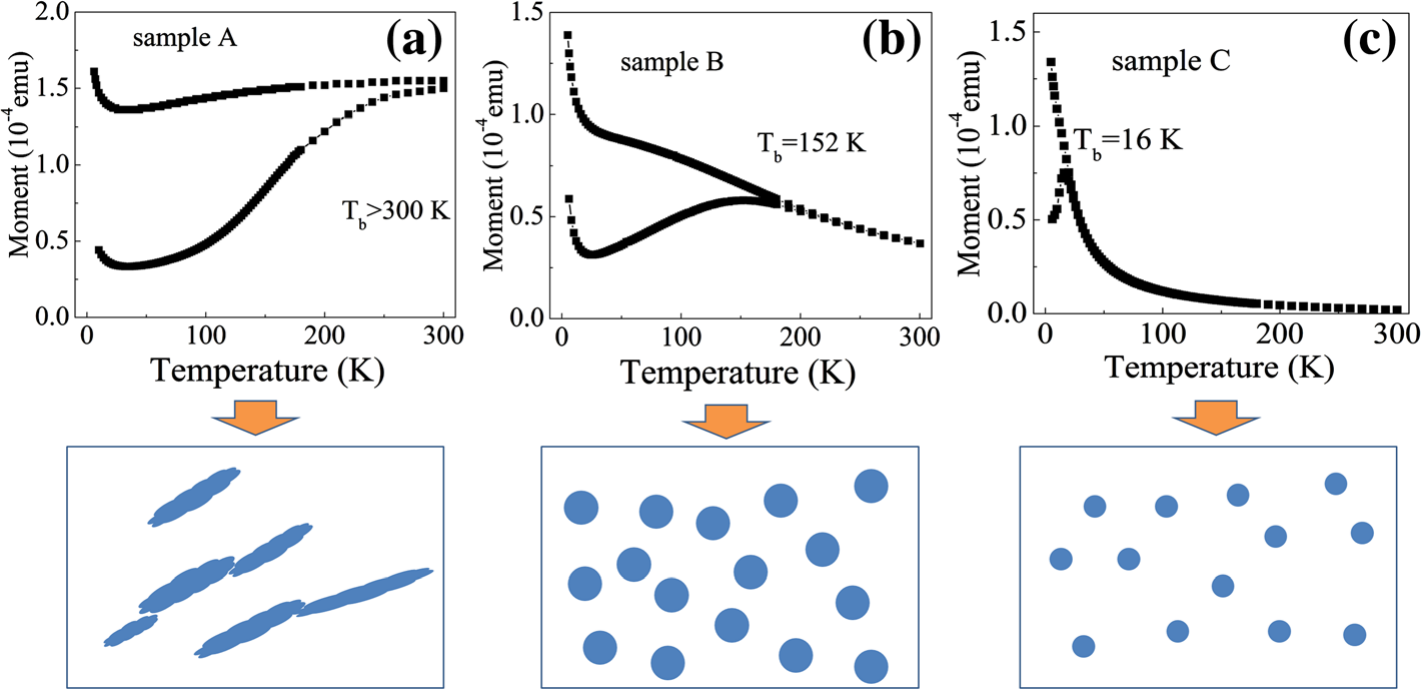
**Temperature dependence of the ZFC-FC curves and plane-view schematic illustrations of the three films. (a)** Sample A, **(b)** sample B, and **(c)** sample C.

Figure 3a,b,c shows the temperature dependence of the zero-field-cooled and field-cooled (ZFC-FC) curves for samples A, B, and C measured in an applied field of 100 Oe. A large bifurcation is observed at low temperatures between the ZFC and FC curves for samples B and C, which suggests that superparamagnetic nanoparticles are embedded in the ZnO matrix [16,17]. Assuming that interactions between Co particles are neglected for samples B and C, the Co particle size can be roughly estimated from the measured blocking temperatures (*T*_*b*_) identified by the maximum in the ZFC plots using the Bean-Livingston formula: *KV =* 25*k*_*B*_*T*_*b*_, where *K* = 2.7 × 10^5^ J/m^3^ is the magnetic anisotropy constant, *V* is the average volume of the nanoparticles, and *k*_*B*_ is the Boltzmann constant. The average size values are approximately 7.2 and 3.4 nm calculated for sample B (*T*_*b*_ = 152 K) and sample C (*T*_*b*_ = 16 K), respectively. However, for sample A, the ZFC and FC plots do not coincide at temperatures below 300 K. This observation is consistent with the ferromagnetic behavior as shown in the inset of Figure 2. The existence of Co nanoparticles and their different dispersion in the ZnO is expected to significantly influence the MR behavior, as will be discussed later.

**Figure 4:**
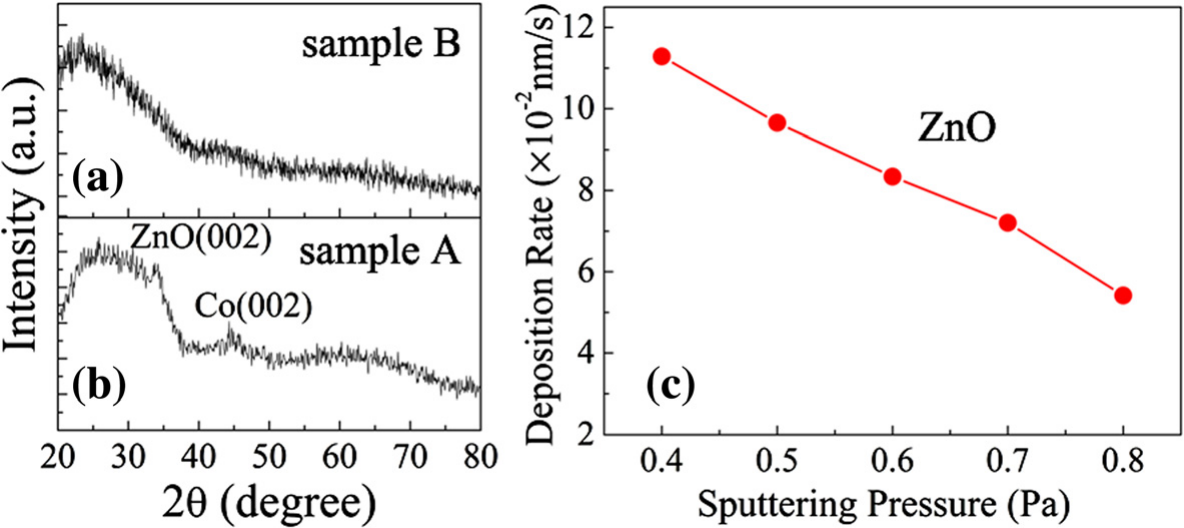
**XRD patterns and variation of deposition rate with sputtering pressure.** XRD pattern of **(a)** sample A and **(b)** sample B. **(c)** Deposition rate of ZnO film.

We also carried out XRD measurements for samples A and B, as shown in Figure 4a,b. Sample B exhibits no peaks because of the small Co particles and amorphous ZnO. Broadened peaks of Co (002) and ZnO (002) appear in sample A, although the Co content of sample A is lower than that of sample B according to the nominal structure of the films. This finding indicates that the distribution of Co particles is inhomogeneous in sample A. Figure 4c shows the variation of the deposition rate of ZnO film with sputtering pressure. The deposition rate decreases from 0.113 to 0.054 nm/s with an increase in sputtering pressure from 0.4 to 0.8 Pa, which is attributed to the increase in collisions and the scattering of sputtered species under high processing pressure [18,19]. In general, the surface of the ZnO film deposited at low pressure is very rough, and a ravine-like topography can form at the surface because of higher deposition rate [18,20]. In our experiments, Co does not wet the surface of ZnO when Co deposits on the surface of ZnO. Co consequently may agglomerate into larger elongated particles in ravines because the surface energy of metallic Co (approximately 2.52 J/m^2^) is higher than that of ZnO (approximately 1.58 J/m^2^). For sample C, superparamagnetic Co particles with smaller size and larger distance between Co particles may form because of the increase in ZnO content and higher sputtering pressure.

From the above discussions, it can be concluded that the films of samples B and C contain Co nanoparticles with different particle sizes dispersed in the ZnO matrix, and some interconnected Co particles may exist in sample A. The plane-view schematic illustrations of the three samples are shown in Figure 3. The structural, magnetic, and transport measurements strongly suggest that the MR effect in these granular films should be related to the size and spatial distribution of Co particles. In the metallic regime, the value of MR decreases with decreasing resistivity probably because of the increase in the number of interconnected Co particles. When the resistivity is less than 0.004 Ω · cm, the value of MR is almost zero. Most Co particles connect with one another and provide few opportunities for spin-polarized electron tunneling. The MR ratio is also reduced as the resistivity in the hopping regime increases, but it still remains greater than 3.7% even when resistivity reaches 3.8 Ω · cm and the volume fraction of Co calculated according to the nominal structure of Co (0.6)/ZnO (2.0) is less than 24%. This observation can be ascribed to the relatively long spin-coherence length in our material [21,22].

We turn to the spin polarization of electron in the films, which can be estimated roughly from the Inoue-Maekawa model as follows: MR = *P*^2^*m*^2^/(1 + *P*^2^*m*^2^) [23], where *P* is the spin polarization of the tunneling electrons, *m* is the relative magnetization of the film, and *m*^2^ = 〈 cos *θ*〉. *m* = 1 in the saturated state, and the above equation becomes MR = *P*^2^/(1 + *P*^2^). The RT spin polarization in the tunneling regime calculated from the MR value of 8.1% is approximately 30%, which is very close to the 35% of the bulk Co metal determined by tunneling [24]. This large RT spin polarization indicates that the transport of polarized carriers in the semiconductor ZnO is very efficient in our films.

**Figure 5:**
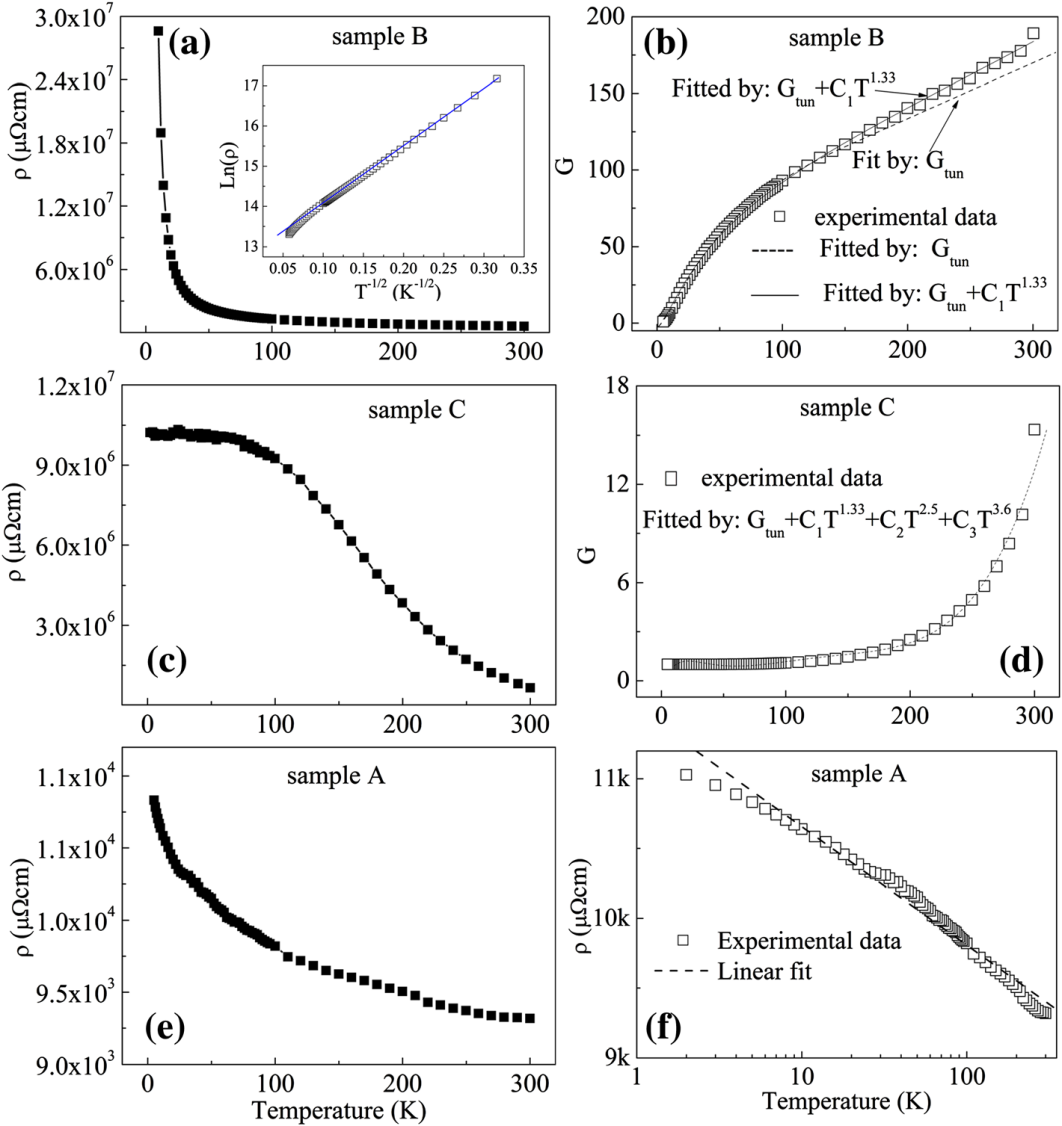
**Relationship between electrical resistivity and temperature. (a)** Electrical resistivity as a function of temperature for sample B. The inset shows the dependence of ln *ρ* on *T*^−1/2^; the solid line represents the linear fit result. **(b)** Illustrations of the theoretical fits of conductivity as a function of temperature for sample B obtained from Equations 1 and 2. **(c)** Electrical resistivity as a function of temperature for sample C. **(d)** Conductivity as a function of temperature for sample C; dotted line is the fitting curve obtained from Equation 2. **(e)** Electrical resistivity as a function of temperature for sample A. **(f)** Electrical resistivity vs logarithmic temperature for sample A.

We focus on the electron transport properties in different regimes. We begin by discussing the intermediate regime (tunneling regime). Figure 5a shows the temperature dependence of the resistivity of sample B, which attests to a semiconductor behavior. As shown in the inset of Figure 5a, from the ln *ρ* vs *T*^−1/2^ plot, it can been seen that ln *ρ* is almost linear to *T*^−1/2^, which is a typical characteristic of interparticle spin-dependent tunneling in metal/insulator granular films [25,26]. To investigate the transport mechanism further, we convert the temperature dependence of resistivity to the temperature dependence of conductivity (*G*), as shown in Figure 5b. The data were normalized to the conductivity at *T* = 5 K. For *T* < 130 K, the interparticle tunneling conductivity of sample B as a function of temperature can be fitted well by the following equation [23,27]:

(1) $$G_{\mathrm{tun}}=G_{0}\exp\left[-{\left(\Delta /T\right)}^{1/2}\right],$$

where *G*_tun_ is the tunneling conductivity, *G*_0_ is a free parameter, Δ *=* 4*E*/*k*_*B*_, *E* is the tunneling activation energy, and *k*_*B*_ is the Boltzmann constant. That is, the ZnO matrix behaves as a tunneling barrier between Co nanoparticles, and the MR effect originates from interparticle spin-dependent tunneling. When *T* > 130 K, the conductivity starts to deviate slightly from Equation 1. This phenomenon suggests that *G*_tun_ is not the only conduction mechanism at high temperature, which may result from the essential physics of the conductance in the presence of localized states within the ZnO matrix. A power-law temperature dependence of conductivity, which is a characteristic of higher-order inelastic hopping, can be used at high temperature to fit the experimental data of sample B. The expression is as follows [28]:

(2) $$G=G_{\mathrm{tun}}+G_{\mathrm{hop}}=G_{0}\exp\left[-{\left(\Delta /T\right)}^{1/2}\right]+CT^{\gamma },$$

where *G*_0_ and *C* are free parameters, *γ* = *N* − *[N*/(*N* + *1*)*]*, *N* is the number of localized states in the barriers, and *G*_hop_ is the spin-independent higher-order inelastic hopping conductivity. Equation 2 fits our experimental data well with *γ* = 1.33 (*N* = 2) at high temperatures, as shown in Figure 5b. At high temperature, the conduction in sample B mainly contains two channels: the tunneling channel and the second-order hopping. The suppression of spin-dependent contribution to the conductance can result in a decrease in the MR at high temperature when a spin-independent channel (i.e., higher-order inelastic hopping) influences the conductivity.

Figure 5c shows the temperature dependence of the resistivity of sample C located in the hopping regime. At low temperatures, an almost temperature-independent tunneling regime is observed. The direct tunneling may represent an important contribution to the total conductance at low temperature, which is similar to the result reported by de Moraes et al. [29]. Figure 5d shows the temperature dependence of the conductivity of sample C and the curve fitted by Equation 2. It is obvious that not only the second-order hopping (*γ* = 1.33) but also the third-order hopping (*γ* = 2.5) and fourth-order hopping (*γ* = 3.6) evidently become non-negligible because a thicker ZnO barrier results in spin-independent higher-order inelastic hopping (see Figure 3c). In order to compare the fitting results of the tunneling and hopping regimes, the resulting parameters fitted by Equation 2 for samples B and C are given in Table 1. It can be seen that the number of localized states of sample C (*N* = 4) increases as compared to sample B (*N* = 2). Consequently, a much higher-order hopping gradually prevails during the transition from the tunneling regime to the hopping regime, which apparently suppresses the MR effect at RT (shown in Figure 1). Also, the tunneling activation energy (*E*) estimated from Δ is 1.64 meV for sample B. With the ZnO content increasing, the value appreciably increases to 44.3 meV due to smaller Co particles and thicker ZnO barriers between Co particles, which consists with the decrease of MR effect in the hopping regime with more defects.

**Table 1:** Fitting results and mainly transport mechanism of three samples

	**Sample 1**	**Sample 2**	**Sample 3**
Applied model	Equation 2	Equation 2	Linear fit
*N*	2	4	-
*G*_0_ (S · cm^−1^)	219.1	31.2	-
*C*_1_ (S · cm^−1^ · K^−1.33^)	3.1 × 10^−2^	8.2 × 10^−3^	-
*C*_2_ (S · cm^−1^ · K^−2.5^)	-	4.0 × 10^−4^	-
*C*_3_ (S · cm^−1^ · K^−3.6^)	-	6.1 × 10^−8^	-
∆ (K)	104.7	2,832.4	-
*E* (meV)	1.64	44.35	-
Straight slope (μΩ · cm/log(K))	-	-	−849.1
Mainly transport	Tunneling	Hopping	Metallic paths

The temperature dependence of conductivity of samples B and C are fitted by Equation 2, as shown in Figure 5b,d. The relationship between resistivity and ln *T* for sample A is fitted linear in Figure 5f.

For sample A, the resistivity as a function of temperature is shown in Figure 5e. Although the temperature coefficient of resistivity is negative below RT, the temperature dependence of resistivity between sample A and the others exhibits evident differences. The resistivity increases gradually with decreasing temperature and varies slightly from 0.0093 Ω · cm (*T* = 300 K) to 0.011 Ω · cm (*T* = 5 K). Combined with the structure of sample A, the transport process is probably dominated by metallic paths because of the large number of interconnected elongated Co particles (see Figure 3a), which decreases when the resistivity increases, accompanying an increased MR effect. The approximate linear relationship between *ρ* and ln *T* for sample A is shown in Figure 5f. The fitting value of straight slope is shown in Table 1. The same phenomenon was reported in a CoO-coated monodispersive Co cluster system corresponding to a small negative MR value in a metal/semiconductor transition regime [30] and in the CoFeB/MgO films, in which the sample with high magnetic metal concentration is not in the strongly localized regime of conduction and the resistivity is plotted as a linear function of log(*T*) [31]. Further detailed studies are necessary and in progress to elucidate the mechanism behind this result.

## Conclusions

In summary, the structure, magnetic properties, and MR effect were investigated in Co/ZnO films deposited by sputtering at different pressures with different ZnO contents. We observed that the MR effect is strongly related to the resistivity of the films. Based on conduction, the MR effect can be classified into three regimes: the metallic, tunneling, and hopping regimes. Large RT MR values greater than 8.1% were obtained in the tunneling regime with a range of resistivity from 0.08 to 0.5 Ω · cm. By contrast, the MR value decreases distinctly when the resistivity of the films is less than 0.08 Ω · cm (metallic regime) or greater than 0.5 Ω · cm (hopping regime). In the tunneling regime, the conduction of the films mainly has two channels: the spin-dependent tunneling channel, which gives rise to high RT MR effect, and the spin-independent second-order hopping (*N* = 2). In the hopping regime, the increased spin-independent higher-order hopping (*N* > 2) through the localized states in thicker ZnO matrix served an important function and is the main reason for the rapid decrease in tunneling MR. In the metallic regime, metallic paths between interconnected elongated Co particles impede the MR effect. These results facilitate a deeper understanding of the spin transport mechanism in metal/semiconductor granular films and are significant for the improvement of the RT MR effect in spintronic applications.

## Competing interests

The authors declare that they have no competing interests.

## Authors’ contributions

Z-YQ designed and performed the experiment, analyzed the results, and drafted the manuscript. LZ and WL performed the tests on the samples and helped perform the experiment. HZ helped in interpreting the transport properties of films. X-HX supervised the work and revised the manuscript. All authors read and approved the final manuscript.
